# Prediction of Gut Microbial Community Structure and Function in Polycystic Ovary Syndrome With High Low-Density Lipoprotein Cholesterol

**DOI:** 10.3389/fcimb.2021.665406

**Published:** 2021-07-19

**Authors:** Xuping Zhu, Yanyu Li, Yanmin Jiang, Jisheng Zhang, Ru Duan, Lin Liu, Chao Liu, Xiang Xu, Lu Yu, Qian Wang, Fan Xiong, Chengming Ni, Lan Xu, Qing He

**Affiliations:** ^1^ Department of Endocrinology, The Affiliated Wuxi People’s Hospital of Nanjing Medical University, Nanjing Medical University, Wuxi, China; ^2^ Department of Good Clinical Practice (GCP), The Affiliated Wuxi People’s Hospital of Nanjing Medical University, Wuxi, China

**Keywords:** gut microbiota, low-density lipoprotein cholesterol, polycystic ovary syndrome, disorder of glucose and lipid metabolism, subtype

## Abstract

Gut microbiota has been proved to be involved in the occurrence and development of many diseases, such as type 2 diabetes, obesity, coronary heart disease, etcetera. It provides a new idea for the pathogenesis of polycystic ovary syndrome (PCOS). Our study showed that the gut microbial community of PCOS with high low-density lipoprotein cholesterol (LDLC) has a noticeable imbalance. Gut microbiota of PCOS patients was significantly changed compared with CON, and these changes were closely related to LDLC. Gut microbiota may affect the metabolic level of PCOS patients through multiple metabolic pathways, and lipid metabolism disorder may further aggravate the imbalance of gut microbiota. *Actinomycetaceae*, *Enterobacteriaceae* and *Streptococcaceae* had high accuracy in the diagnosis of PCOS and the differentiation of subgroups, suggesting that they may play an important role in the diagnosis and treatment of PCOS in the future. Also, the model we built showed good specificity and sensitivity for distinguishing PCOS from CON (including L_CON and L_PCOS, H_CON and H_PCOS). In conclusion, this is the first report on the gut microbiota of PCOS with high LDLC, suggesting that in the drug development or treatment of PCOS patients, the difference of gut microbiota in PCOS patients with different LDLC levels should be fully considered.

## Introduction

Polycystic ovary syndrome (PCOS) is one of the most common anovulatory infertility in women of childbearing age (Zhao et al., 2016; Persson et al., 2019). The prevalence rate of PCOS in women of childbearing age is as high as 5–10%, accounting for 50–70% of anovulatory infertility (O’Brien and Emans, 2008). PCOS is characterized by excessive androgen secretion, ovulation disorder and polycystic ovarian changes, accompanied by abdominal obesity, insulin resistance, impaired glucose metabolism and dyslipidemia (Zhang et al., 2019).

At present, it has been confirmed that gut microbiota can interact with the body, which plays a vital role in food digestion, energy metabolism, immune regulation and gene expression. The changes in abundance and structure may be the potential pathogenic mechanism of a variety of diseases. At present, several studies have shown that gut microbiota disorder is closely associated with insulin resistance, type 2 diabetes, obesity, coronary heart disease, metabolic syndrome and other disorders of glucose and lipid metabolism (Fujisaka et al., 2016; Pedersen et al., 2016; Lindheim et al., 2017), which provides a new idea for the pathogenesis of polycystic ovary syndrome.

In recent years, many studies have shown that the gut microbiota of PCOS patients changes. These changes are closely related to obesity, BMI, insulin resistance (IR) and so on (Insenser et al., 2018; Zeng et al., 2019; Jobira et al., 2020). There are also some reports about the importance of lipid metabolism in the pathogenesis of PCOS (Ghaffarzad et al., 2016; Göbl et al., 2016; O’Reilly et al., 2017). However, so far, there is no relevant literature on the relationship between gut microbiota and blood lipid changes in PCOS patients. In the previous analysis of the data, our team found that there was a close correlation between the changes of gut microbiota in PCOS patients and low-density lipoprotein cholesterol (LDLC), and LDLC was most related (*R^2^ = 0.195*, *P = 0.001*) to the changes of gut microbiota in subjects (**Table 1**).

**Table 1:** Relationship between sample distribution and clinical indicators(Redundancy analysis).

	RDA1	RDA2	R^2^	*P*
LDLC	0.6614	−0.75	0.195	***0.001***
GLU 30 min	0.9858	−0.1682	0.1466	***0.002***
GLU 2 h	0.8843	−0.467	0.1675	***0.002***
HOMA IR	0.4904	−0.8715	0.1131	***0.008***
INS 2 h	0.8866	−0.4625	0.1106	***0.009***
ApoB/ApoAI	0.8451	−0.5346	0.1003	***0.013***
SBP	0.2214	−0.9752	0.0917	***0.018***
INS 30 min	0.8606	−0.5092	0.0917	***0.018***
WHR	0.8372	−0.547	0.0777	***0.036***
TG	0.6447	−0.7644	0.0759	***0.04***
DBP	0.1418	−0.9899	0.058	*0.077*
BMI	0.449	−0.8935	0.0555	*0.09*
FSH	−0.8107	−0.5854	0.0384	*0.2*
P	0.8797	−0.4755	0.0367	*0.207*
PRL	0.9497	0.3133	0.0288	*0.276*
Ts	0.8741	−0.4858	0.0253	*0.342*
GLU 0 h	0.6558	−0.7549	0.0146	*0.548*
ApoA1	−0.418	−0.9084	0.0114	*0.642*
Age	0.6233	0.782	0.0059	*0.783*
E2	0.9995	−0.0305	0.006	*0.798*
LH	0.6628	−0.7488	0.0039	*0.839*
HDLC	−0.2484	0.9687	0.0008	*0.962*
AMH	−0.8381	−0.5455	0.0009	*0.969*

The bold part in the table represents statistics, P < 0.05, with statistical significance.

In our study, we analyzed the different structural and functional characteristics of gut microbiota in CON and PCOS patients at diffident LDLC levels. Here, we identified several essential gut microbiota in patients with PCOS and evaluated their correlation with clinical metabolic parameters, the possibility of diagnostic typing, and functional prediction analysis. These findings may contribute to the improvement of blood lipids, especially improving LDLC in gut microbiota-based drug design and clinical treatment of polycystic ovary syndrome.

## Materials And Methods

### Study Participants

A total of 54 premenopausal women aged 18–35 years were with a definite diagnosis of PCOS were collected from the Department of Endocrinology of Wuxi People’s Hospital Affiliated to Nanjing Medical University, the Endocrinology Department of Jiangyin People’s Hospital and the Department of Gynecology of Wuxi Maternal and Child Health Care Hospital during June 2018 to June 2020. The samples of this study were all from women of childbearing age in Southeast Asia. The healthy control group was composed of 33 women of premenopausal age who underwent physical examination in the physical examination center of Wuxi People’s Hospital at the same time, with ordinary blood lipid, PCOS and chronic intestinal diseases excluded.

In this study, the diagnosis of PCOS was based on the Rotterdam criteria revised by the Dutch conference in 2003 (Fauser, 2004). Individuals with one of the following conditions were excluded: ① pregnancy or other diseases affecting hyperandrogenemia and abnormal glucose and lipid metabolism, androgen-secreting tumor, adrenal disease, thyroid insufficiency, Cushing’s syndrome, smoking, diabetes mellitus; ② within 12 weeks before the evaluation of the first screening stage of this study received Diane-35, metformin, antibiotics, other estrogen and progesterone, lipid-lowering, hypoglycemic, glucocorticoid and other drug treatment; ③ There was liver function impairment (alt, AST >1.5 times of the upper limit laboratory average population index), chronic liver disease.

This study protocol was reviewed and approved by the ethics committee of Wuxi People’s Hospital (ethical batch number: 2017-IIT-08-01) and has passed the Chinese clinical trial registration (Registration Number: ChiCTR1800016346) (Ni et al., 2020). All participants in this study signed informed consent of the protocol.

### Anthropometric and Metabolic Parameter Measurements

All subjects were examined in the morning after a night’s fasting (>8 h). For healthy women or PCOS patients without amenorrhea, blood samples were collected 2–4 days during menstruation. However, for PCOS patients with amenorrhea, blood samples were collected on any day of the menstrual cycle. About 3–5 g of fresh feces were collected from each participant during the non-menstrual period and transferred to the laboratory’s refrigerator within 2 h (keeping the temperature below 4°C), and then frozen at −80°C until detection.

The waist circumference (WC) was measured in the middle between the lower sternum edge and the iliac crest. The hip circumference (HC) was measured at the maximum hip extension level. The subjects had to take a rest at least 5 min before. Laboratory biochemical indexes were measured by an automatic biochemical analyzer (Beckman AU5800 clinical chemistry system, Beckman Coulter, Inc., South Kraemer Boulevard, Brea, CA, USA). Blood glucose and insulin were measured on an empty stomach or after 75 g glucose diluted in 250–300 ml water for half an hour and 2 h later (OGTT).

WHR = WC (cm)/HC (cm)

BMI = weight (kg)/height2 (m^2^).

Lipoprotein ratio: TG (mmol/L)/HDL-C (mmol/L)

HOMA-IR = GLU 0 h (mmol/L) ∗ INS 0 h (mmol/L)/22.5 (Long et al., 2019)

LAP = [WC (cm) − 58] × TG (mmol/L) (Wiltgen et al., 2009)

VAI (females) = [WC (cm)/(36.58 + 1.89 ∗ BMI)] ∗ (TG (mmol/L)/0.81) ∗ (1.52/HDLC (mmol/L)) (Brończyk-Puzoń et al., 2017)

CVAI (females) = −187.32 + 1.71 ∗ age + 4.23 ∗ BMI + 1.12 ∗ WC (cm) + 39.76 ∗ Log10TG (mmol/L) − 11.66 ∗ HDLC (mmol/L) (Long et al., 2019)

### Grouping of Subjects

All subjects completed the baseline assessment. The control (CON) group consisted of 18 healthy women with the regular menstrual cycle, ordinary blood lipid, and with no signs of Hyperandrogenemia (HA) or polycystic ovary (PCO). Approximately 33 CON and 54 PCOS patients were included in the study according to the inclusion criteria. According to the “Guidelines for prevention and treatment of dyslipidemia in Chinese adults” (Joint Committee on Revision of guidelines for prevention and treatment of dyslipidemia in Chinese adults, 2016), the stratification standard of dyslipidemia in primary prevention population of ASCVD in China, LDLC ≥3.4 was regarded as the standard of abnormal increase of LDLC. Approximately 15 CON were divided into high LDLC (H_CON) group according to LDLC ≥3.4mmol/L, and the remaining 18 CON with LDLC <3.4 mmol/L were divided into low LDLC (L_CON) group. Approximately 16 PCOS patients were divided into high LDLC (H_PCOS) group according to LDLC ≥3.4 mmol/L, and the remaining 38 PCOS patients with LDLC <3.4 mmol/L were divided into low LDLC (L_PCOS) group.

### Fecal DNA Extraction and Sequencing

According to the manufacturer’s instructions, the total DNA was extracted from each fecal sample using the QIAamp DNA fecal micro Kit (Qiagen 51504, Germany) and treated with RNase A at 37°C for 7 min (final concentration of 0.1 mg/ml) before digestion by proteinase K digestion. DNA concentration was determined by a nanodrop spectrophotometer and adjusted to 10 ng/ml. The v3–v4 regions of the 16S rRNA gene were amplified by universal primers 338F (50-ACT CCT ACG GGA GGC AGC AG-30) and 806R (50-GGA CTA CHV GGG TWT CTA AT-30). PCR amplification was performed in a 20 ml mixture containing 4 μl of 5×FastPfu buffer, 2 μl of 2.5 mM dNTPs, 0.8 μl of each primer (5 μM), 0.4 μl of FastPfu Polymerase (TransGen Biotech, Beijing, China), and 10 ng of template DNA. The amplification procedure included the initial denaturation step (94°C for 5 min), 27 amplification cycles (94°C 30 s, 55°C 30 s and 72°C 30 s) and the last extension at 72°C for 10 min (ABI GeneAmp 9700, USA) (Zeng et al., 2019). After 2% agarose gel electrophoresis, AxyPrep DNA Gel Extraction Kit (Axygen Biosciences, Union City, CA) was used to extract the amplified products and then quantified by QuantiFluor™-ST system (Promega, Fitchburg, WI). Purified amplicons were pooled at equimolar concentration and pair-end sequenced on Illumina Miseq PE300 platform (Illumina, San Diego, Ca) (Liu et al., 2017; Zhou et al., 2020) according to the standard protocols (Majorbio Bio-Pharm Technology, Shanghai, China).

### Statistical Analysis

#### Clinical Data Statistical Analysis

The clinical data were calculated by SPSS statistical software package 20.0. The data of normal distribution were analyzed by variance (three groups and above), and the two groups were compared by *t-test*, expressed as *mean ± standard deviation*. The data of non-normal distribution were analyzed by *nonparametric Wilcoxon test* and expressed by a *median with interquartile range (IQR)*. Spearman correlation analysis was used to analyze the correlation between blood lipid and gut microbiota. All statistical tests were double-tailed. When *p <0.05*, the difference was statistically significant.

#### Bioinformatics Analysis

As previously described, Trimmmatic was used to control the quality of the original sequencing data. According to a similarity threshold of 97%, the OTUs were clustered by Uparse V7.1 (http://drive5.com/uparse/), and the representative sequences of OTUs were obtained by removing chimeras (Cole et al., 2014). Extracting non-repetitive sequences from the optimized sequences can reduce the number of redundant calculation in the analysis process. To obtain the species classifications information of each OTU, RDP classifier V2.2 (http://sourceforge.net/projects/rdp-classifier/) was used to classify 97% OTU representative sequences (Cole et al., 2014). The default confidence threshold was 70%.

The rarefaction estimates were calculated using QIIME v1.9.1 (Caporaso et al., 2010). The curve graph was made using the R language tool. Shannon index, sobs index and ace index were calculated in Mothur built-in commands to reveal the Alpha diversity of gut microbial community (Schloss et al., 2009). In order to test the significance of the difference of gut microbial community (βeta diversity) among groups, 999 iterations were performed by analysis of similarity (ANOSIM) based on Bray Curtis faith distance (Li et al., 2017). The Kruskal–Wallis test compared the relative abundance of four bacteria groups, and the error detection rate was controlled by the Tukey–Kramer post-hoc test method (Driscoll, 1996). The Spearman correlation coefficient was used to evaluate the potential correlation between gut microbial community and clinical parameters. The variance expansion factor (VIF) was used to reduce the collinearity of clinical indicators. Redundancy analysis (RDA) multiple regression analysis was used to rank the clinical indicators and gut microbiota.

Receiver Operating Characteristic (ROC) Curve was made using the R language (plot ROC package) to reveal the relationship between sensitivity and specificity of gut microbiota. The Kyoto Encyclopedia of Genes and Genomes (KEGG) database (http://www.genome.jp/kegg/) and the Phylogenetic Investigation of Communities by Reconstruction of Unobserved States (PICRUSt) were used to predict the functional profile of gut microbial communities (Langille et al., 2013). The number of each KEGG gene was calculated, and the abundance of the KEGG pathway was estimated at each KEGG level.

## Results

### The Differences of Alpha Diversity and Beta Diversity Between CON and PCOS Groups

Based on the v3–v4 region of the 16S rRNA gene, the imbalance of gut microbial community was studied by the second-generation sequencing. A total of 4,473,516 high-quality sequences were obtained from 87 samples. The optimized base number was 1,834,170,957 bp, and the average length of the optimized sequence was 410.01.

**Figure 1:**
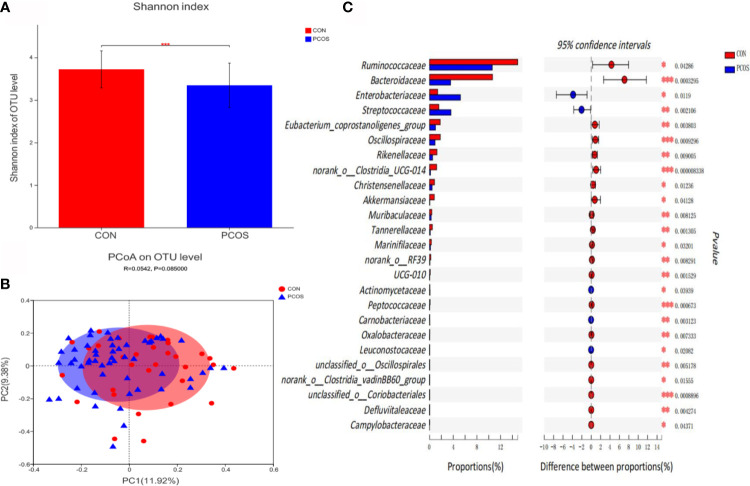
Comparative analysis of gut microbiota between CON and PCOS groups. **(A)** The Shannon index of OTU level showed the difference between CON and PCOS groups. **(B)** Principal coordinate analysis (PCoA) of gut microbiota was based on *Bray Curtis faith distance* (ANOSIM, *P = 0.085, R = 0.0.0542*). Each point represents the bacterial community composition of a single fecal sample, and the axis title represents the percentage change of interpretation (11.92% for PC1 and 9.38% for PC2). **(C)** 25 groups of gut microbiota with statistical difference between CON and PCOS were screened out. *P < 0.05, **P < 0.01, ***P < 0.001.

Bioinformatics analysis showed that the composition and distribution of gut microbiota in PCOS and CON changed significantly. Shannon index (**Figure 1A**) suggested α diversity of the gut microbiota in PCOS patients was significantly lower than that of CON group (*P <0.05*). The results of Principal coordinate analysis (PCoA) of gut microbiota (**Figure 1B**) showed that PCOS patients were different from CON group, but they could not be separated accurately and clearly (*P ＞0.05*). At the family level, there were 25 species of gut microbiota with significant differences between groups. Compared with CON, *Ruminococcaceae*, *Bacteroidaceae*, *Eubacterium_coprostanoligenes_group*, *Oscillospiraceae*, *Rikenellaceae*, *norank_o:Clostridia_UCG-014*, *Christensenellaceae*, *Akkermansiaceae*, *Muribaculaceae*, *Tannerellaceae*, *Marinifilaceae*, *norank_o:RF39*, *UCG-010*, *Peptococcaceae*, *Oxalobacteraceae*, *unclassified_o:Oscillospirales*, *norank_o:Clostridia_vadinBB60_group*, *unclassified_o:Coriobacteriales*, *Defluviitaleaceae* and *Campylobacteraceae* decreased significantly in PCOS, while *Enterobacteriaceae*, *Streptococcaceae*, *Actinomycetaceae*, *Carnobacteriaceae* and *Leuconostocaceae* increased significantly in PCOS (**Figure 1C**).

### Gut Microbiota Correlations With Clinical Indicators

**Figure 2:**
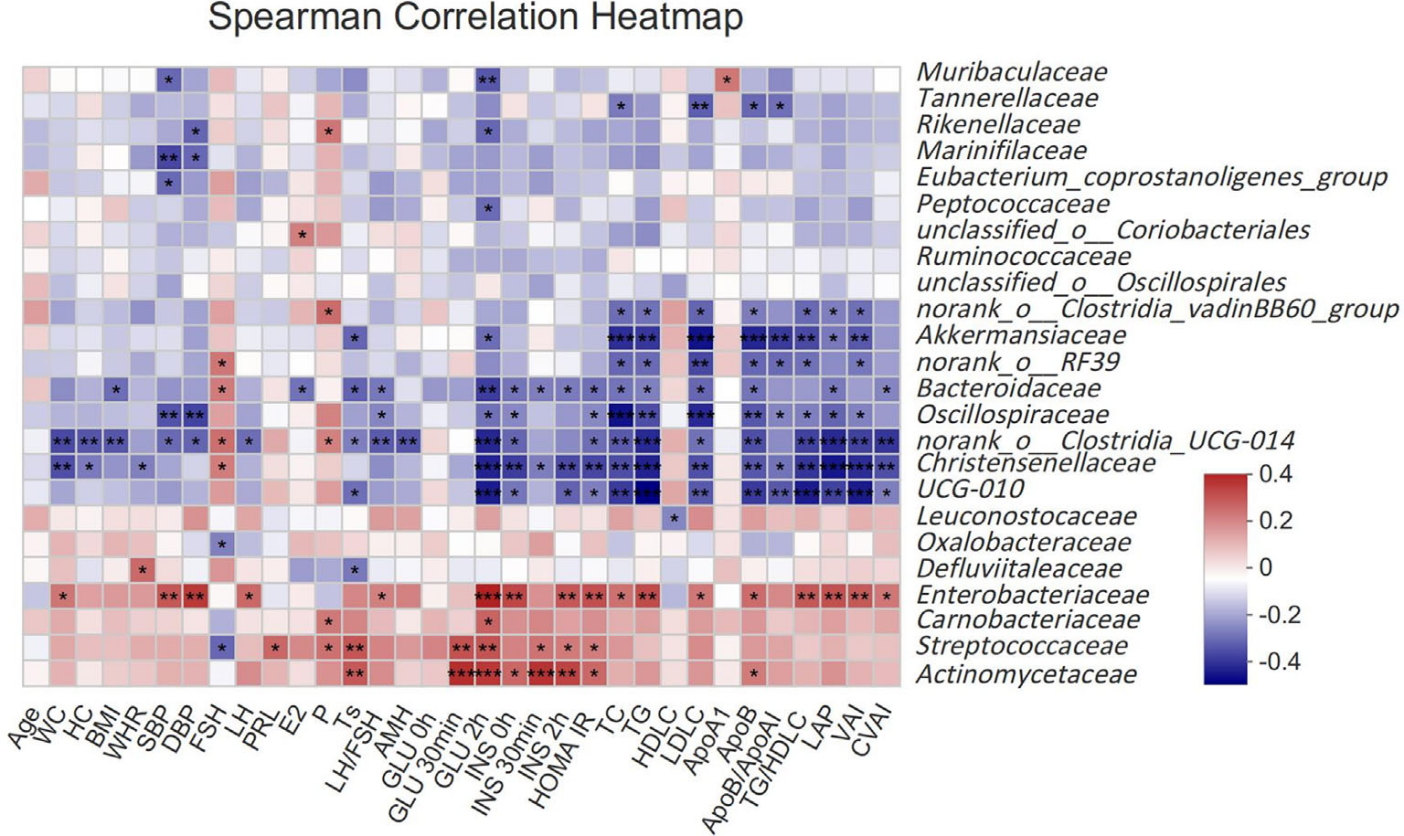
Spearman correlation thermogram of clinical indicators and gut microbiota at Family level. Blue indicated a negative correlation and red indicated a positive correlation. The depth of the color represented the strength of the correlation. The deeper the color was, the stronger the correlation was. *P < 0.05, **P < 0.01, ***P < 0.001.

To assess the relationship between gut microbiota and clinical indicators, we calculated the correlation between the 25 different gut microbiota mentioned above and clinical indicators in subjects (**Figure 2**). *Akkermansiaceae*, *norank_o:RF39*, *Bacteroidaceae*, *Oscillospiraceae*, *norank_o:Clostridia_UCG-014*, *Christensenellaceae* and *UCG-010* were negatively correlated with the most related indexes of glucose and lipid metabolism such as GLU, INS, HOMA IR, TC, TG, LDLC, ApoB, TG/HDLC, LAP, VAI, CVAI, etcetera (*P ＜0.05*), while *Enterobacteriaceae* had a strong positive correlation with those indexes. *Streptococcaceae* and *Actinomycetaceae* had a stronger correlation with glucose metabolism (GLU, INS, HOMA IR), but a weaker correlation with lipid metabolism. *Akkermansiaceae*, *Bacteroidaceae*, *norank_o:Clostridia_UCG-014*, *UCG-010* and *Defluviitaleaceae* were negatively correlated with Ts, while *Streptococcaceae* and *Actinomycetaceae* were positively correlated with Ts.

### The Explanatory Power of Clinical Indicators on the Difference of Gut Microbiota

In order to reduce the influence of multiple collinear relationship between clinical indicators, we used the variance expansion factor (VIF) to screen several times until the VIF value of the selected clinical indicators was less than 10 (**Supplementary Table 1**). Then we used Redundancy analysis (RDA) multiple regression analysis to rank the clinical indicators and gut microbiota (**Table 1**). As shown in **Table 1**, LDLC, GLU 30 min, GLU 2 h, HOMA IR, INS 2 h, ApoB/ApoAI, SBP, INS 30 min, WHR and TG was statistically significant in explaining the difference of gut microbiota distribution. It showed that the disorder of glucose and lipid metabolism was an important reason for the difference of gut microbiota between CON and PCOS. LDLC was the most relevant clinical index (*R^2^ = 0.195, P = 0.001*).

### General Measurements and Metabolic Parameters of Subjects at Different LDLC Levels

To further explore the correlation between LDLC and gut microbiota of CON and PCOS, we divided CON into H_CON and L_CON groups and divided PCOS patients into H_PCOS and L_PCOS groups according to “Guidelines for prevention and treatment of dyslipidemia in Chinese adults” (Joint Committee on Revision of guidelines for prevention and treatment of dyslipidemia in Chinese adults, 2016).

After adjusting for Age and BMI, the differences of clinical indexes among the four groups were analyzed, **Table 2**. It showed that FSH, LH, Ts, LH/FSH, AMH had significant differences between CON and PCOS groups, mainly manifested as increased LH, Ts, LH/FSH, AMH and decreased FSH (*P <0.05*), which means that disorder of sex hormones in PCOS patients. There was no significant difference in the subgroup of CON and PCOS (*P >0.05*). GLU 2h, INS 0h, INS 2h, HOMA IR were mainly manifested increase in H_PCOS group, but there was no statistical difference among the other three groups, suggesting that the glucose metabolism of PCOS patients with high LDLC was significantly disordered, such as increased postprandial blood glucose, hyperinsulinemia and insulin resistance. TC, TG, LDLC, ApoB, ApoB/ApoA1, CVAI also had significant differences between CON and PCOS, suggesting that PCOS (especially H_PCOS) had obvious disorder of lipid metabolism (*P <0.05*).

**Table 2:** Anthropometric and metabolic parameters of all participants.

	L_CON	H_CON	L_PCOS	H_PCOS	*P*	*P-adjusted*
Age	27.28 ± 3.82	27.53 ± 3.82	25.74 ± 3.26	26.75 ± 4.16	*0.298*	*/*
WC	73.08 ± 6.49	75.36 ± 7.07	83.82 ± 11.18	87.09 ± 15.37	***＜0.001***	*0.342*
HC	91.89 ± 5.31	89.17 ± 8.36	98.54 ± 10.06	102.19 ± 14.07	***0.001***	*0.084*
BMI	20.72 ± 2.23	21.74 ± 2.51	25.06 ± 4.67	26.75 ± 8.27	***0.001***	*/*
WHR	0.79 ± 0.05	0.85 ± 0.06	0.84 ± 0.06	0.85 ± 0.06	***0.017***	*0.074*
SBP	110.61 ± 10.37	114 ± 11.43	117.03 ± 11.25	123.75 ± 12.37	***0.009***	*0.211*
DBP	67.89 ± 7.02^cd^	69.73 ± 8.24^c^	75.66 ± 7.35^ab^	76.06 ± 8.67^a^	***0.001***	***0.016***
FSH	8.8 ± 2.97^cd^	8.1 ± 1.55	6.37 ± 1.75^a^	6.03 ± 2.52^a^	***＜0.001***	***0.015***
LH	5.44 ± 1.91^cd^	5.31 ± 2.88^cd^	10.67 ± 6.62^ab^	9.07 ± 6.13^ab^	***0.001***	***0.005***
PRL	13.55 ± 10.28	15.43 ± 10.06	13.61 ± 6.18	13.22 ± 5.71	*0.856*	*0.777*
E2	35.50 (24.75,50.00)	35.00 (23.00,51.00)	52.50 (37.75,65.00)	49.00 (32.25,73.50)	***0.008***	*0.053*
P	0.47 (0.34,0.73)	0.49 (0.36,0.68)	0.56 (0.28,0.99)	0.62 (0.36,1.19)	*0.808*	*0.118*
Ts	35.52 ± 15.55^cd^	45.69 ± 25.5^cd^	71.01 ± 24.86^ab^	71.05 ± 24.56^ab^	***＜0.001***	***＜0.001***
LH/FSH	0.68 ± 0.35^cd^	0.66 ± 0.36^cd^	1.65 ± 0.92^ab^	1.54 ± 0.75^ab^	***＜0.001***	***＜0.001***
AMH	3.94 ± 2.29^cd^	2.96 ± 1.7^cd^	10.5 ± 5.67^ab^	9.8 ± 5.09^ab^	***＜0.001***	***＜0.001***
GLU 0 h	4.93 ± 0.25	5.22 ± 0.32	5.19 ± 0.45	5.2 ± 0.33	*0.064*	*0.188*
GLU 30 min	6.92 ± 0.97	7.21 ± 1.12	7.55 ± 1.33	7.88 ± 1.21	*0.105*	*0.267*
GLU 2 h	5.63 ± 0.76^cd^	6.46 ± 1.14^cd^	7.47 ± 1.65^ab^	7.94 ± 1.41^ab^	***＜0.001***	***＜0.001***
INS 0 h	7.3 ± 3.39^d^	9.14 ± 5.35^d^	14.8 ± 8.61^d^	21.81 ± 13.46a^bc^	***＜0.001***	***0.01***
INS 30 min	51.52 ± 32.18	46.78 ± 21.79	85.6 ± 78.92	116.74 ± 68.64	***0.005***	*0.232*
INS 2 h	42.41 ± 21.03^d^	44.74 ± 26.58^d^	103.83 ± 76.51^d^	158.1 ± 118.74^abc^	***＜0.001***	***0.008***
HOMA IR	1.61 ± 0.79^d^	2.15 ± 1.29^d^	3.46 ± 2.11^d^	5.14 ± 3.5^abc^	***＜0.001***	***0.023***
TC	4.03 ± 0.41^bcd^	5.75 ± 0.67^ac^	4.61 ± 0.71^ab^	5.96 ± 0.77^ac^	***＜0.001***	***＜0.001***
TG	0.76 ± 0.29^bd^	1.37 ± 1.07^a^	1.35 ± 0.64	1.73 ± 0.77^a^	***0.002***	***0.042***
HDLC	1.39 ± 0.18	1.43 ± 0.31	1.21 ± 0.29	1.27 ± 0.24	***0.022***	*0.259*
LDLC	2.09 ± 0.37^bcd^	3.77 ± 0.26^ac^	2.69 ± 0.49^abd^	4.07 ± 0.71^ac^	***＜0.001***	***＜0.001***
ApoA1	1.48 ± 0.2	1.52 ± 0.27	1.49 ± 0.39	1.52 ± 0.31	*0.972*	*0.972*
ApoB	0.59 ± 0.09^bcd^	0.94 ± 0.16^acd^	0.79 ± 0.18^abd^	1.14 ± 0.26^abc^	***＜0.001***	***＜0.001***
ApoB/ApoAI	0.41 ± 0.08^bd^	0.64 ± 0.16^a^	0.57 ± 0.21^d^	0.79 ± 0.27^ac^	***＜0.001***	***＜0.001***
TG/HDLC	0.56 ± 0.23	1.03 ± 0.9	1.24 ± 0.75	1.45 ± 0.79	***0.002***	*0.141*
LAP	11.76 ± 6.59	26.7 ± 31.51	39.19 ± 30.56	52.25 ± 37.51	***0.001***	*0.255*
VAI	1.02 ± 0.45	1.86 ± 1.57	2.35 ± 1.48	2.73 ± 1.51	***0.002***	*0.134*
CVAI	6.73 ± 18^bcd^	23.38 ± 28.93^a^	45.85 ± 40.43^a^	62.24 ± 56.37^a^	***＜0.001***	***0.018***

All data except E2 and P were normal distribution, and were analyzed by ANOVA, expressed as mean ± SD. E2 and P of non-normal distribution were analyzed by nonparametric Wilcoxon test and expressed as median with interquartile range (IQR). ^a^P < 0.05 for statistically differently from L_CON. ^b^P < 0.05 for statistically differently from H_CON. ^c^P < 0.05 for statistically differently from L_PCOS. ^d^P < 0.05 for statistically differently from H_PCOS. P-adjusted: adjusted for Age and BMI. The bold part in the table represents statistics, P < 0.05, with statistical significance.

### Alpha Diversity and Beta Diversity of Gut Microbial Communities at Different LDLC Levels

**Figure 3:**
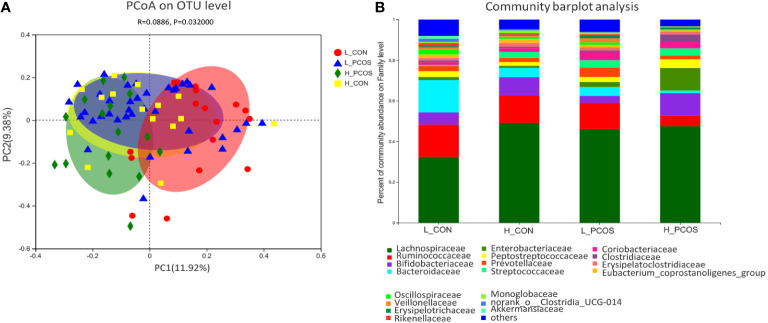
Principal coordinate analysis and Community barplot analysis of the gut microbial communities. **(A)** Principal coordinate analysis (PCoA) of gut microbiota was based on Bray–Curtis faith distance (ANOSIM, *P = 0.032, R = 0.0886*). Each point represents the bacterial community composition of a single fecal sample, and the axis title represents the percentage change of interpretation (11.92% for PC1 and 9.38% for PC2). **(B)** Community barplot analysis at Family level in each group.

PCoA based on Bray–Curtis faith distance revealed that the L_CON and H_PCOS could be well separated. In contrast, the H_CON and L_PCOS were mixed between the two groups and could not be distinguished (**Figure 3A**). The gut microbiota with high LDLC subgroup, either CON or PCOS, shifted to the abnormal direction, suggesting that the composition of gut microbiota in high LDLC group changed, and LDLC was an important factor in the change of gut microbiota (**Table 1**, RDA: *R^2^ = 0.195, P = 0.001*). However, when people with high LDLC were compared (even if there was no difference in LDLC between H_CON and H_PCOS), the gut microbiota of H_PCOS still deviated to the abnormal direction, suggesting that there were other confounding factors affecting the composition of gut microbiota at the same time. Community barplot analysis showed that *Lachnospiraceae*, *Ruminococcaceae*, *Bifidobacteriaceae*, *Bacteroidaceae*, *Streptococcaceae* and *Oscillospiraceae* had significant differences among groups (**Figure 3B**).

**Figure 4:**
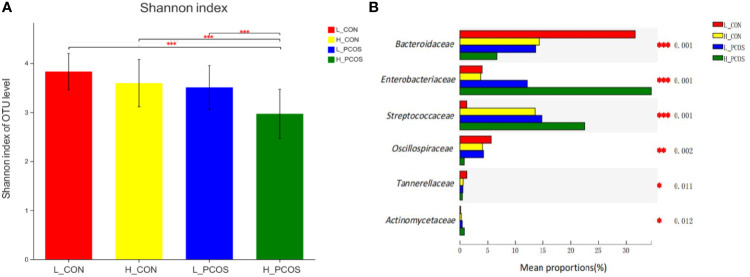
Alpha diversity and Beta diversity of the gut microbial communities after adjustment for Age and BMI. **(A)** The Shannon index of OTU level showed the difference among the four groups. **(B)** Six groups of gut microbiota with statistical difference among the four groups were screened out. *P < 0.05, **P < 0.01, ***P < 0.001.

After adjusting for Age and BMI, Shannon index (**Figure 4A**) suggested α diversity of the gut microbiota in H_PCOS was lowest among the four groups, while Shannon index in L_CON was highest (*P <0.05*). α diversity of gut microbiota in H_PCOS decreased significantly (*P <0.05*), while α diversity had a tendency to decrease in H_CON and L_PCOS compared with that in CON (*P ＞0.05*).

After adjusting for Age and BMI, six groups of gut microbiota with statistical difference among the four groups were screened out in **Figure 4B**. Compared with L_CON, *Enterobacteriaceae*, *Streptococcaceae* and *Actinomycetaceae* had a significant increase in H_PCOS, while *Bacteroidaceae*, *Oscillospiraceae* and *Tannerellaceae* had a significant decrease in H_PCOS.

### ROC Analysis for Identifying Disease Status and Subtypes by Gut Microbiota Characteristics

**Figure 5:**
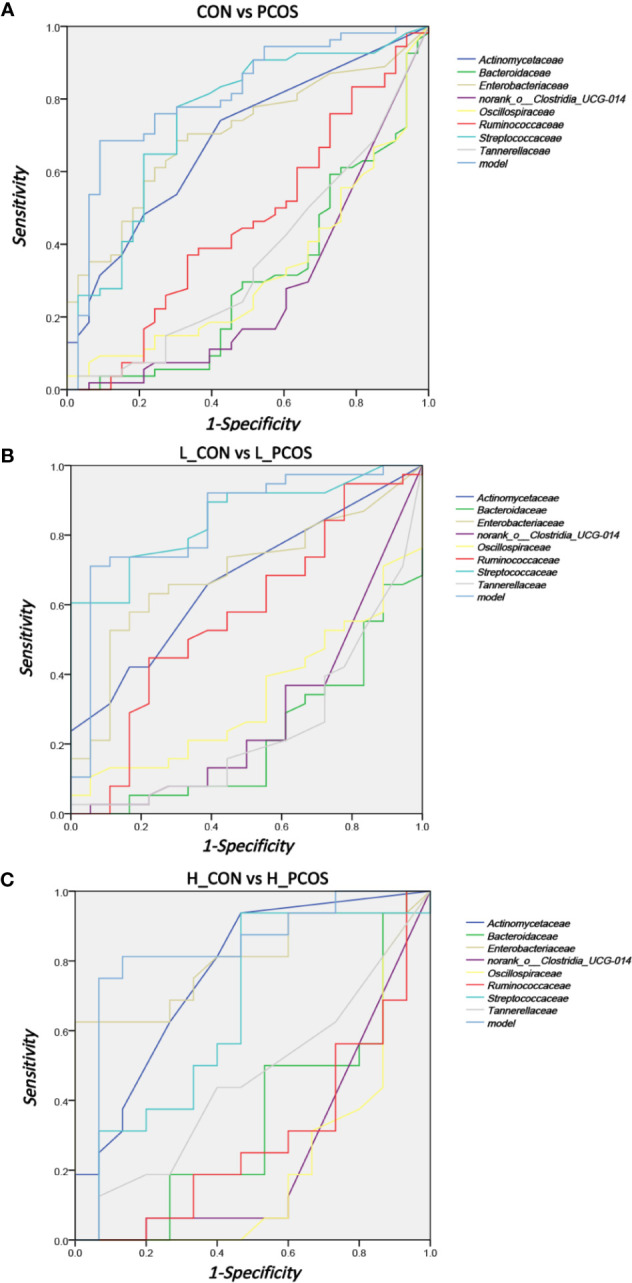
ROC analysis for identifying disease status and subtypes by gut microbiota characteristics. **(A)** ROC analyses of 8 different gut microbiotas and the model were used to evaluate the ability of distinguishing PCOS. **(B, C)** ROC analysis of eight different gut microbiotas and the model were used to evaluate the ability of distinguishing L_CON and L_PCOS,H_CON and H_PCOS.

Receiver operating curve (ROC) analysis was performed to explore the association of important related taxa with PCOS diagnosis status and subgroup grouping at the Family level (**Figure 5**). Area under ROC curve (AUC), sensitivity and specificity of each taxon were shown in **Table 3**. *Actinomycetaceae*, *Enterobacteriaceae* and *Streptococcaceae* were found to have good performance in distinguishing PCOS from CON (including L_CON and L_PCOS, H_CON and H_PCOS). The AUC value of *Streptococcaceae* used to distinguish CON from PCOS was *0.747 (95% CI 0.636,0.857) (P ＜0.001)*, and to distinguish L_CON from L_PCOS was *0.847 (95% CI 0.748, 0.947) (P ＜0.001)*. The AUC value of *Enterobacteriaceae* used to distinguish H_CON from H_PCOS was *0.804 (95% CI 0.644, 0.964) (P = 0.004)*.

**Table 3:** ROC correlation analysis.

	CON *vs* PCOS		L_CON *vs* L_PCOS		H_CON *vs* H_PCOS	
	***AUC (95%CI)***	***P***	***AUC (95%CI)***	***P***	***AUC (95%CI)***	***P***
*Actinomycetaceae*	***0.689 (0.576, 0.802)***	***0.003***	***0.671 (0.527, 0.815)***	***0.04***	***0.769 (0.599, 0.938)***	***0.011***
*Bacteroidaceae*	*0.306 (0.189, 0.423)*	*0.003*	*0.219 (0.092, 0.347)*	*0.001*	*0.35 (0.15, 0.55)*	*0.155*
*Enterobacteriaceae*	***0.705 (0.597, 0.814)***	***0.001***	***0.691 (0.548, 0.834)***	***0.022***	***0.804 (0.644,0.964)***	***0.004***
*Oscillospiraceae*	*0.327 (0.21, 0.443)*	*0.007*	*0.336 (0.193, 0.48)*	*0.049*	*0.213 (0.041,0.384)*	*0.006*
*Streptococcaceae*	***0.747 (0.636, 0.857)***	***＜0.001***	***0.847 (0.748, 0.947)***	***＜0.001***	***0.663 (0.461, 0.864)***	***0.123***
*Tannerellaceae*	*0.36 (0.24, 0.481)*	*0.029*	*0.245 (0.108, 0.382)*	*0.002*	*0.463 (0.256, 0.669)*	*0.722*
*Ruminococcaceae*	*0.46 (0.332, 0.588)*	*0.532*	*0.569 (0.401, 0.736)*	*0.41*	*0.296 (0.108, 0.483)*	*0.053*
*norank_o_Clostridia_* *UCG-014*	*0.282 (0.166, 0.398)*	*0.001*	*0.286 (0.135, 0.437)*	*0.01*	*0.25 (0.07, 0.43)*	*0.018*
*model*	***0.81 (0.714, 0.906)***	***＜0.001***	***0.839 (0.722, 0.956)***	***＜0.001***	***0.829 (0.669, 0.99)***	***0.002***

The bold part in the table represents statistics, P < 0.05, with statistical significance.

Furthermore, we used logistic regression to analyze the difference of 25 kinds of bacteria between PCOS and CON groups step by step, and established the prediction model according to the regression coefficient, the expression was: −0.009 ∗ *Bacteroidaceae* − 0.04 ∗ *norank_o:Clostridia_UCG014* − 0.006 ∗ *Ruminococcaceae* + 4.042. The model showed good specificity and sensitivity for distinguishing PCOS from CON (including L_CON and L_PCOS, H_CON and H_PCOS).The AUC value of the model to distinguish CON from PCOS was *0.81 (95% CI 0.714, 0.906) (P ＜0.001)*, to distinguish L_CON from L_PCOS was *0.839 (95% CI 0.722, 0.956) (P ＜0.001)*, and to distinguish H_CON from H_PCOS was *0.829 (95% CI 0.669, 0.99) (P = 0.002)*.

### Prediction and Analysis of the Metabolic Function of Characteristic Gut Microbiota

In this study, the metabolic function of gut microbiota was enriched and predicted based on KEGG level-3. Approximately 371 KEGG metabolic pathways were screened out, 30 of which had significant differences (*P <0.05*). After adjusting for Age and BMI, the 30 KEGG metabolic pathways still had significant differences among groups.

It showed that there were significant differences in KEGG function prediction among the four groups, mainly in energy metabolism (Citrate cycle (TCA cycle), Ubiquinone and other terpenoid-quinone biosynthesis), steroid metabolism (Steroid hormone biosynthesis), inflammation (Lipopolysaccharide biosynthesis, Arachidonic acid metabolism), nucleotide metabolism (Glycosphingolipid biosynthesis—ganglio series, Riboflavin metabolism, Lipoic acid metabolism), lipid metabolism (Adipocytokine signaling pathway), glycometabolism (glycan degradation), protein metabolism (Protein digestion and absorption), apoptosis and autophagy (Lysosome, Peroxisome, Apoptosis).

We found that the activity of steroid metabolism, lipid metabolism, apoptosis and autophagy pathway were significantly increased in PCOS patients, especially in group H_PCOS group. Energy metabolism, inflammation and other pathways can be observed to be significantly enriched in the population with high LDLC (both H_CON and H_PCOS).

## Discussion

At present, PCOS is considered to be a multi-gene regulation and multi-factor induced disease. Its pathogenesis is still unclear, with significant heterogeneity, different clinical treatment effects and many complications. Its short-term complications include infertility, abortion, preterm birth, gestational diabetes and other adverse pregnancy outcomes. In the long term, it can increase the risk of diabetes, endometrial cancer, cardiovascular and cerebrovascular diseases, and even digestive tract tumors and other diseases (Lindheim et al., 2017). In recent years, clinicians pay more and more attention to the occurrence of glucose and lipid metabolism disorders in PCOS patients. Studies show that about 70% of PCOS patients have insulin resistance and glucose and lipid metabolism disorders, whether fat or thin. After improving the metabolic disorders, some patients’ conditions have been alleviated (Merkin et al., 2011; Palomba et al., 2014; Echiburú et al., 2016). O’Reilly et al. found that the concentrations of glycerophospholipids (GPL) and lysophospholipids (LGPL) were significantly increased in women with PCOS, but not in the control group (O’Reilly et al., 2017). Both GPL and LGPL have been considered as markers of risk and progression of nonalcoholic fatty liver disease (NAFLD) (Anjani et al., 2015). O’Reilly et al. further demonstrated that inter adipose androgen excess and dysfunctional lipid metabolism were causal drivers of low metabolic risk in patients with PCOS (O’Reilly et al., 2017). Some studies have shown that hypomethylated genes related to lipid and steroid synthesis may promote the synthesis of steroids, including androgens, which may partly explain the mechanism of hyperandrogenemia in PCOS (Pan et al., 2018).

The number of the human gut microbiota genome is more than 100 times that of the human genome, and it has been given the title of “new organ of human body”. In recent years, the gut microbiota has become a research hotspot. According to the research findings, 2,172 different kinds of bacteria have been isolated from the human feces, which can be divided into 12 different phyla, of which 93.5% belong to Proteobacteria, Firmicutes, Actinobacteria and Bacteroidetes (Li et al., 2014; Hugon et al., 2015). Bacteroides and Firmicutes in PCOS patients were changed compared with those in ordinary people (Liu et al., 2017). The α-diversity was decreased, and the β-diversity and metabolites of the gut microbiota were changed in PCOS (Su et al., 2015). Torres et al. proved that the decrease of α-diversity in PCOS patients was negatively correlated with the level of total testosterone and hirsutism. In addition, hyperandrogenemia was closely related to the β-diversity of the gut bacterial community (Torres et al., 2018). In PCOS patients, *Bacteroidaceae*, *Porphyromonadaceae*, *Clostridiaceae*, *Erysipelotrichidae*, *Lachnospiraceae*, *Lactobacillaceae* and *Ruminococcaceae* in Firmicutes were changed compared with ordinary people. Because of these changes in gut microbiota, butyrate and propionate production decreased, affecting the integrity of the intestinal barrier and immunity in PCOS patients (Su et al., 2015). Sherma et al. found that the number of *Akkermansia*, *Bacteroides*, *Lactobacillus* and *Clostridium* decreased in PCOS patients. Administration of *Akkermansia* can improve the intestinal barrier function (Sherman et al., 2018). Qi et al. found that gut microbiota *B. vulgatus* in PCOS patients led to increased ovarian function damage, insulin resistance, bile acid metabolism changes, which was mediated by the decrease of IL-22 secretion. By regulating GATA binding protein 3 to induce IL-22 secretion, PCOS phenotype can be improved. These results suggest that the changes of intestinal microflora, bile acid metabolism and/or IL-22 level may be valuable for the treatment of PCOS (Qi et al., 2019).

Current studies have shown that the gut microbiota may affect the disease progression of PCOS through the following ways: ① by destroying the function of tight junction and increasing the intestinal permeability, resulting in the increase of plasma lipopolysaccharide (LPS) level, which mediates chronic inflammation *in vivo* (Witta et al., 2008); ② the decrease of short chain fatty acid (SCFA) level can increase the infiltration of macrophages in adipose tissue, and destroy the intestinal tight junction by reducing the production of mucus It can increase intestinal permeability and induce insulin resistance (Peng et al., 2007); ③ the change of intestinal bile acid can cause the decrease of intestinal glucagon like peptide-1 (GLP-1) secreted by intestinal L cells and induce metabolic abnormalities *in vivo*, thus affecting the reproductive axis (Thomas et al., 2009); ④ it can increase the occurrence of insulin resistance and mediate the disorder of glucose and lipid metabolism by increasing the concentration of branched chain amino acids (Qiao et al., 2013).

In the previous analysis of our data, our team found a close correlation between the changes of gut microbiota in PCOS patients and LDLC, and LDLC was most related (*R^2^ = 0.195, P = 0.001*) to the changes of gut microbiota in subjects (**Table 1**).

We used PCoA cluster analysis to cluster group L_CON, H_CON, L_PCOS and H_PCOS (**Figure 3A**). It showed the group L_CON and the group H_PCOS could be well separated, while group H_CON and L_PCOS were mixed between the two groups and could not be well differentiated. This indicated that the gut microbiota of PCOS patients with low LDLC has changed compared with that of ordinary people, and the gut microbiota has changed to H_ LDLC direction offset. Also, it indicated that when people with high LDLC were compared (even if there was no difference in LDLC between H_CON and H_PCOS), the gut microbiota of H_PCOS still deviated to the abnormal direction, suggesting that there were other confounding factors affecting the composition of gut microbiota at the same time. These may be caused by insulin resistance, sex hormone changes, or other dyslipidemia. However, there was no doubt that LDLC played an important role in the distribution of gut microbiota in both CON and PCOS.

Seyam et al. found a significant increase in LDLC in PCOS, which increased cardiovascular disease risk (Liu et al., 2019). Seyam et al. further found a decrease in LDLC and improved the symptoms after statin treatment (Seyam et al., 2018). In a multivariate analysis of the non-PCOS population, Shelley et al. found that androgen levels were positively correlated with LDL in women but not in women with higher BMI (Shelley et al., 1998). A large number of studies have proved that oral lipid-lowering drugs and probiotics can improve the structure and function of gut microbiota, and ultimately improve the disorder of host lipid metabolism. Liu et al. found that rosuvastatin can improve the structure of gut microbiota and dyslipidemia (Martinez-Guryn et al., 2018). Kaddurah et al. found that simvastatin can reduce blood lipid by affecting the synthesis of secondary bile acids by intestinal microorganisms (Kaddurah-Daouk et al., 2011). It has been reported that androgen could down-regulate LDLC receptor function *in vivo* and *in vitro* (Crook and Seed, 1990; Croston et al., 1997). Dyslipidemia was associated with increased androgen levels in PCOS (Fruzzetti et al., 2009) and was corrected by antiandrogen therapy (Diamanti-Kandarakis et al., 1998). Gut microbiota can affect the level of serum LDLC in mice by up regulating the expression of LDLC receptor in the liver. The absence of gut microbiota in sterile mice can lead to the increase of serum LDLC (Kasahara et al., 2017). Shen et al. found that *Akkermansiaceae* protected the host from hyperlipidemia by enhancing the expression of LDLC receptor and apolipoprotein E (Apo-E) (Shen et al., 2016). Thus, the gut microbiota can reduce the risk of cardiovascular disease by affecting the host LDL receptor signaling pathway.

We further analyzed the differences among the groups (**Figure 4B**). Compared with L_CON, *Enterobacteriaceae*, *Streptococcaceae* and *Actinomycetaceae* had a significant increase in H_PCOS. Human studies have reported an increased abundance of *Enterobacteriaceae* in obesity (Karlsson et al., 2012; Fei and Zhao, 2013). Lindberg et al. found that LPS of *Enterobacteriaceae* showed significantly higher endotoxin activity than LPS of other gut microbiota (Lindberg et al., 1990). As well, it was found that *Enterobacteriaceae* was positively correlated with reproductive hormones such as Ts, AMH and LH (Zheng et al., 2021). Jobira et al. also found that *Streptococcaceae* increased in the gut microbiota of PCOS patients, which was consistent with our results (Jobira et al., 2020). *Streptococcaceae* has been proved to be closely related to the development of obesity (Wei et al., 2020). *Streptococcaceae* was found to be helpful to metabolism and absorption of carbohydrate in intestine (Sheridan et al., 2016), leading to the increase of blood glucose and blood lipid levels. At the same time, this imbalance may lead to mucosal immune imbalance, lymphocyte activation, enhanced intestinal permeability (Quraishi et al., 2017), the infiltration of macrophages in fat tissue increases, and further destroys the intestinal tight connection by reducing the production of mucus, which makes the inflammatory factors such as lipopolysaccharide increase in blood, induce insulin resistance (Peng et al., 2007), and form a vicious cycle. Adams et al. found that *Actinomycetaceae* increased significantly in patients with nonalcoholic fatty liver disease (Adams et al., 2020). *Actinomycetaceae* were found to be involved in liver fat synthesis and promote lipid accumulation (Ge et al., 2020).

As well, *Bacteroidaceae* and *Oscillospiraceae* and *Tannerellaceae* were found to decrease most significantly in group H_PCOS. *Oscillospiraceae* and *Bacteroidaceae* were found to be negatively correlated with cholesterol (Maya-Lucas et al., 2019), while probiotics could promote the production of SCFA, regulate the composition of gut microbiota, and improve the intestinal barrier function (Liu et al., 2020). It was consistent with our study results using Spearman correlation to describe the relationship between lipid metabolism and gut microbial communities.

It was found that the *Tannerellaceae* of mice fed with high-fat diet increased significantly (Farzi et al., 2021). *Tannerellaceae* family is most abundant in some gastrointestinal diseases, such as Crohn’s disease (Dash et al., 2019), suggesting its importance in immune regulation.

We further studied the functional characteristics of the gut microbial community by PICRUST analysis. As shown in the KEGG related metabolic pathways in **Table 4**, the metabolic pathways such as Steroid hormone biosynthesis, Glycosphingolipid biosynthesis—ganglio series, Apoptosis, Adipocytokine signaling pathway and Protein digestion and absorption were different among the groups. The main manifestation was that metabolism was active in PCOS patients, especially in H_PCOS group.

**Table 4:** Comparison of predicted microbial function among groups based on KEGG level-3.

Pathway	Description	L_CON	H_CON	L_PCOS	H_PCOS	*P*	*P- adjusted*
ko00020	Citrate cycle (TCA cycle)	351,334.21 ± 93,088.22	375,267.58 ± 67,911.43	377,369.5 ± 66,494.99	430,028.61 ± 82,065.37	***0.034***	***0.033***
ko00130	Ubiquinone and other terpenoid-quinone biosynthesis	34,526.93 ± 20,180.95	48,236.93 ± 36,159.37	36,063.45 ± 18,960.04	68,406.79 ± 32,225.79	***0.008***	***0.009***
ko00140	Steroid hormone biosynthesis	1,902.35 ± 1,975.1	4,102.49 ± 6,033.17	4,837.47 ± 4,535.9	10,297.82 ± 9,395.48	***0.002***	***0.001***
ko00511	Other glycan degradation	144,904.05 ± 68,048.27	153,467.28 ± 88,698.64	169,721.32 ± 66,684.76	246,271.38 ± 13,5904.39	***0.007***	***0.009***
ko00513	Various types of N-glycan biosynthesis	8,312.99 ± 8,859.24	14,104.16 ± 23,238.7	18,515.98 ± 14,838.54	42,496.41 ± 41,444.81	***0.001***	***0.001***
ko00531	Glycosaminoglycan degradation	32,594.86 ± 16,207.68	39,364.99 ± 30,565.98	46,072.72 ± 19,797.63	75,394.37 ± 54,474.98	***0.002***	***0.002***
ko00540	Lipopolysaccharide biosynthesis	56,334.52 ± 59,486.34	91,126.53 ± 79,253.52	66,928.89 ± 49,138.02	142,785.22 ± 91,379.04	***0.007***	***0.008***
ko00590	Arachidonic acid metabolism	5,211.34 ± 2,764.32	7,501.44 ± 5,430.82	5,594.83 ± 2,469.28	9,628.47 ± 5,455.28	***0.027***	***0.022***
ko00604	Glycosphingolipid biosynthesis - ganglio series	8,302.37 ± 8,858.24	14,079.21 ± 23,247.47	18,490.61 ± 14,834.72	42,453.23 ± 41,412.3	***0.001***	***0.001***
ko00740	Riboflavin metabolism	13,2418.42 ± 2,4458.5	143,664.06 ± 29,409.83	125,524.33 ± 24,896.48	150,973.54 ± 25,787.97	***0.029***	***0.027***
ko00785	Lipoic acid metabolism	10,587.21 ± 3,990.54	14,503.15 ± 8,553.36	14,403.08 ± 7,078.32	23,124.52 ± 12377.24	***0.001***	***0.002***
ko00908	Zeatin biosynthesis	35,442.97 ± 5,111.3	37,639.82 ± 7,508.75	36,719.57 ± 6,275.03	42,504.96 ± 8,559.48	***0.038***	***0.049***
ko01053	Biosynthesis of siderophore group nonribosomal peptides	4,045.95 ± 2,958.55	9,572.72 ± 11,627.59	3,572.7 ± 2,533.19	10,832.01 ± 7,628.72	***0.016***	***0.012***
ko01503	Cationic antimicrobial peptide (CAMP) resistance	205,541.31 ± 51,410.77	222,532.96 ± 53,104.78	190,477.16 ± 44,313.83	236,928.02 ± 54,443.6	***0.049***	***0.047***
ko02060	Phosphotransferase system (PTS)	460,405.17 ± 214,352.99	391,095.96 ± 146,694.45	360,225.62 ± 167,602.21	296,180.69 ± 125,633.31	***0.045***	***0.025***
ko03320	PPAR signaling pathway	68,539.34 ± 18,818.87	70,927.33 ± 17,449.07	70,787.96 ± 9,132.27	85,797.59 ± 20,608.98	***0.019***	***0.023***
ko03450	Non-homologous end-joining	612.19 ± 696.09	538.06 ± 573.22	1,232.45 ± 1,560.55	1,356.94 ± 1,614.89	***0.029***	***0.039***
ko04013	MAPK signaling pathway—fly	5,418.28 ± 5,442.76	5,933.47 ± 4,919.29	4,858.82 ± 3,257.66	10,086.93 ± 6,412.82	***0.018***	***0.024***
ko04068	FoxO signaling pathway	9,902.37 ± 10,611.4	9,512.97 ± 5,676.52	10,496.08 ± 5,280.76	16,815.88 ± 10,008.32	***0.017***	***0.033***
ko04142	Lysosome	52,296 ± 29,498.63	59,661.53 ± 56,236.71	71,959.35 ± 36,525.66	128,795.1 ± 96,956.34	***0.001***	***0.002***
ko04146	Peroxisome	10,0250.35 ± 23,899.75	103,899.81 ± 24,115.14	99,440.14 ± 16,858.23	120,604.81 ± 27,035.13	***0.044***	***0.043***
ko04210	Apoptosis	2,206.12 ± 3,013.92	4,445.64 ± 6,456.33	5,235.78 ± 4,715.21	13,374.69 ± 11,417.36	***＜0.001***	***＜0.001***
ko04211	Longevity regulating pathway	8,164.21 ± 10,435.72	7,334.6 ± 5,666.61	7,132.95 ± 4,564.13	13,891.03 ± 9,499.09	***0.026***	***0.044***
ko04216	Ferroptosis	35,938.12 ± 16,259.08	37,104 ± 15,914.56	37,556.59 ± 9,167.76	54,686.8 ± 22,288.58	***0.003***	***0.005***
ko04514	Cell adhesion molecules (CAMs)	0.88 ± 1.35	0.43 ± 0.75	0.07 ± 0.23	1.48 ± 2.99	***0.035***	***0.027***
ko04614	Renin-angiotensin system	542.91 ± 771.08	939.64 ± 1,300.61	1,550.94 ± 1,577.5	2,811.35 ± 2,865.73	***0.001***	***0.002***
ko04714	Thermogenesis	30,227.71 ± 13,677.49	31,484.67 ± 12,765.77	32,695.21 ± 8,205.37	45,272.45 ± 17,189.61	***0.004***	***0.006***
ko04920	Adipocytokine signaling pathway	31,188.21 ± 12,221.78	33,285.76 ± 12,546.05	34,925.26 ± 7,521.56	47,758.91 ± 17,122.07	***0.001***	***0.001***
ko04974	Protein digestion and absorption	3,336.12 ± 3,369.26	6,086.73 ± 8,225.36	6,604.4 ± 4,776	15,587.93 ± 12,651.85	***＜0.001***	***＜0.001***
ko05133	Pertussis	5,475.37 ± 4,094.63	13,662.57 ± 16,738.1	6,694.42 ± 5,299.31	17,946.25 ± 12,034.17	***0.013***	***0.013***

The bold part in the table represents statistics, P < 0.05, with statistical significance.

Excessive steroid synthesis is one of the characteristics of PCOS. In the different gut microbiota prediction of KEGG metabolic pathway, the steroid hormone synthesis pathway was also active in PCOS group, which suggested that the gut microbiota was also involved in androgen metabolism. Yurkovetskiy et al. confirmed that gut microbiota can also negatively regulate plasma testosterone levels, suggesting that gut microbiota changes may be a major cause of abnormal steroid hormone metabolism, and may become an effective treatment (Yurkovetskiy et al., 2013). Poutahidis et al. fed the mice with *L. reuteri*, found that the level of testosterone in the circulating blood of the mice was higher than before (Poutahidis et al., 2014). The team further transplanted the gut microbiota of adult male mice into the intestines of young female mice, and found that the testosterone level of the latter was increased, suggesting that the gut microbiota can change the testosterone level. Another study used probiotic supplementation for 12 weeks in PCOS patients, resulting in significant improvements in hirsutism, total testosterone and SHBG values (Jamilian et al., 2018).

Glucose, lipid metabolism and protein absorption related pathways (Glycosphingolipid biosynthesis—ganglio series, Adipocytokine signaling pathway, Protein digestion and absorption) were significantly active in people with high LDLC, especially in H_PCOS, suggesting that the risk of energy accumulation in PCOS patients was significantly higher than that in healthy people. It is found that the changes of gut microbiota abundance and structure may be the potential pathogenic mechanism of many diseases. The changes of gut microbiota can mediate different nutritional and energy metabolic networks, and play the role of symbiosis and cooperation with the host through this network (Arumugam et al., 2011). At present, a number of studies have shown that there is a close relationship between gut microbiota disorder and excessive energy intake, excessive accumulation of sugar and fat (Fujisaka et al., 2016; Pedersen et al., 2016; Lindheim et al., 2017). This is due to the reduction of short chain fatty acids synthesized by gut microbiota, which participate in insulin mediated lipid accumulation of adipocytes and enhance the absorption of lipids and carbohydrates (Peng et al., 2007).

Apoptosis was also found to be significantly increased in PCOS and correlated with LDLC. It is found that hyperlipidemia can induce the formation of reactive oxygen species, induce oxidative stress, and then mediate cell apoptosis (Li et al., 2016), which was consistent with the results of our study that the apoptotic pathway is active in high LDLC group. Du et al. found that inhibition of gap junction communication can induce apoptosis through mitochondrial pathway (Du et al., 2017). Another study showed that interference with gap junction communication can induce the formation of reactive oxygen species in ovary, and then mediate cell apoptosis (López-Arellano et al., 2019). Gut microbiota has been confirmed by a large number of literatures that it can destroy gap junction, increase intestinal permeability, increase harmful substances into the blood, and cause apoptosis in many ways (Witta et al., 2008).

In this study, we identified several essential characteristic gut microbiotas in patients with PCOS and evaluated their correlation with clinical metabolic parameters. LDLC was most related (*R2 = 0.195, P = 0.001*) to the changes of gut microbiota in subjects, according to which, we further compared the differences of clinical indicators and gut microbiota between CON and PCOS with different LDLC levels. *Actinomycetaceae*, *Enterobacteriaceae* and *Streptococcaceae* were found to have good performance in distinguishing PCOS disease status and subtypes. In PCOS, multiple metabolic pathways were changed, and H_PCOS group had the most active metabolism.

However, this study was a cross-sectional study with a small sample size, which could not be further stratified to exclude confounding factors and verify the underlying mechanism of the interaction between gut microbiota and LDLC in PCOS patients. As well, it may be that PCOS patients are more likely to be obese, and we did not guarantee that the BMI of CON and PCOS was similar, which may also cause the difference of gut microbiota between CON and PCOS and this may become a confounding factor. Therefore, the follow-up study needs to collect CON and PCOS patients with similar BMI. Our research team will further expand the sample size, carry out hierarchical analysis, and regulate the gut microbiota through the use of lipid-lowering drugs, probiotics and other interventions to further study the role of different gut microbiota in the metabolism and hormone levels of PCOS patients, and lay the foundation for further searching for therapeutic targets.

## Conclusion

In conclusion, our study showed that PCOS patients had more serious steroid hormone disorder, glucose and lipid metabolism disorders than CON group, and gut microbiota of PCOS had serious imbalance. After adjusting for Age and BMI, the gut microbiota of PCOS patients was still significantly changed compared with CON, and these changes were closely related to LDLC. Gut microbiota may affect the metabolic level of PCOS patients through multiple metabolic pathways, and lipid metabolism disorder may further aggravate the imbalance of gut microbiota. *Actinomycetaceae*, *Enterobacteriaceae* and *Streptococcaceae* had high accuracy in the diagnosis of PCOS and the differentiation of subgroups, suggesting that they may play an important role in the diagnosis and treatment of PCOS in the future. Also, the model we built showed good specificity and sensitivity for distinguishing PCOS from CON (including L_CON and L_PCOS, H_CON and H_PCOS). In the drug development or treatment of PCOS patients, gut microbiota in PCOS patients with different LDLC levels should be fully considered.

## Data Availability Statement

The data that support the findings of this study are available from the authors on reasonable request. Themouse gut 16S rRNA gene sequencing data was deposited under National Center for Biotechnology Information (NCBI; Bethesda, MD, USA) BioProject PRJNA 734262, and sequence reads are available at NCBI under BioSample IDs SAMN19487522-19487608.

## Ethics Statement

This study protocol was reviewed and approved by the ethics committee of Wuxi People’s Hospital (ethical batch number: 2017-IIT-08-01). The patients/participants provided their written informed consent to participate in this study. Written informed consent was obtained from the individual(s) for the publication of any potentially identifiable images or data included in this article.

## Author Contributions

LX and QH conceived, designed and coordinated the experiments. XZ and YL carried out experiments, drafted and wrote the manuscript. YJ, JZ, RD and LL revised and revised the manuscript. CL, XX and CN analyzed and sorted out the data. LY, QW and FX carried out experiments, collected and sorted out data. All authors contributed to the article and approved the submitted version.

## Funding

This work was supported by the Major Project of Wuxi Health Planning Commission, China (Z201807), General project of Wuxi science and Technology Bureau, China (N20202006), and the Research and practice innovation plan for Postgraduates in Jiangsu Province, China (SJCX20_0491).

## Conflict of Interest

The authors declare that the research was conducted in the absence of any commercial or financial relationships that could be construed as a potential conflict of interest.
